# Annotations

**Published:** 1896-09-05

**Authors:** 


### Annotations.

### The Decline and Fall of the Examiner.

Medical students, who as a class have suffered more than any other persons from the examination craze which has prevailed during the past twenty years, will be glad to see faint streaks in the orient indicating the dawn of a more rational day. The *Times* publishes a letter from Dr. William Ostwald, Professor of Physical Chemistry in the University of Leipsic, in which are set forth at some length various reasons why Germany is so decidedly taking the lead of all other countries, England included, in the pursuit of the more profitable manufactures. The secret of German success, Dr. Ostwald tells us, is the fact that in Germany science always aims at allying itself with practice, and practice, on her part, shows a reciprocal desire to come to terms with science. The point which will interest medical students most in Dr. Ostwald's statement is that which refers to examinations. "The examination," we are told, "varies somewhat in different Universities; but in no case is it either very long or very extensive; indeed it is not considered very important." Now in Great Britain the exact opposite is the case. But in pure and applied science, Germany, with her easy examinations, beats us, with our stiff ones, all along the line. What German University professors have found out, the inspectors in English elementary schools are also finding out; and now they look for signs of mental training where formerly they demanded the fruits of memory. The obvious fact is that what young men, who are really to succeed in life, in medicine as in other pursuits, require, is teaching, not cram; practical training, not book work alone. All the science they are taught should have its practical side. In a busy world, science for science sake is for the few. The best science is that which mends the world. In nothing more than in medical education is it necessary to take a leaf out of Professor Ostwald's book.

### Have Doctors Rights?

That great palladium of British liberty, the jury system, not seldom produces a Paladin who essays, with a courage which is beyond all praise, to set the world right in every detail of its conduct whenever he has the opportunity. At an inquest held the other day at Southwark, it came out in evidence that a doctor who had been sent for had not gone to the case immediately when called, but had sent out a message, although indoors at the time, to say that he was "not at home." The foreman of the jury hearing this, and without waiting to ask what justification the doctor might show, rose in his virtuous indignation, rated the doctor soundly, appealed to the coroner as to what punishment ought to be inflicted on so base a monster, and was followed up by another equally virtuous and equally absurd juryman, who declared that for his part he "should never believe doctors again." Now the doctor probably acted unwisely, and at any rate in a cowardly spirit, who ordered his servant to say he was not at home when he was But it cannot be too clearly understood that a medical man has a perfect right to decline to attend a case whenever he pleases; and if he declines for an adequate reason, as he obviously may do, then no blame attaches to him, and none should be attempted to be put upon him. The two jurymen at Southwark probably did the particular doctor in question a considerable injury by their display of cheap virtue. We call attention to the case in order to make a suggestion. It is this: That jurymen and coroners, who often heap gratuitous insults upon medical men, ought to be compelled to observe a reasonable discretion in their utterances. We suggest that some medical man, who has done all his duty in a case but has failed to satisfy an exacting juryman, shall bring an action for defamation, and so put an end to the practice of heaping undeserved insults upon a hard-worked and often altogether unpaid class of men.

### The Disposal of Town Refuse.

One of the standing difficulties of all town sanitary authorities is the disposal of refuse, and the larger the town the greater the difficulty. Probably few people have any conception of the enormous quantity of dust which is year by year collected, and has by some means or another to be disposed of. From a report made to the County Council a couple of years ago, it appears that not far short of 900,000 tons is collected in London during the year, and as London increases in size not only does the distance which it has to be carried grow longer, but suburban residents grow more particular than they used to be, and object to their select neighbourhoods being used as dumping grounds for other people's rubbish, so that the difficulty of disposing of it constantly increases. Every year then the necessity for destroying it on the spot becomes more urgent, and it is matter for no small surprise that the sanitary authorities of the metropolis are so slow in taking measures for its destruction by fire, as is so commonly done in provincial towns, where from their much smaller size the necessity for such a proceeding would seem infinitely less urgent than it is in London. The objections are partly the expense of land (the expense of the actual cremation is certainly no greater than that of carting the stuff away), and partly the fear of creating nuisance in carrying out the process. The fact is that the use of destructors has got for itself a somewhat bad name in consequence of the very imperfect furnaces which were at first used for the purpose; but we should have to think far more lightly than we now do of the mechanical ingenuity of the age in which we live before we could believe that the wit of man could not devise a means of burning up this mass of rubbish on the spot without producing a nuisance. The production of smell and the emission of dust are entirely the result of deficient combustion, and too great hurry in the process. But the evidence collected for the guidance of the County Council and the improvements which have even since then been made in the machines, as shown in the proposals made by Dr. Macadam before the Scottish Society of Arts, make it clear that all nuisance can be avoided, and it is to be hoped that before long the London authorities will seriously undertake this most necessary duty of burning up the refuse of the town directly it is collected from the houses, as is done in so many provincial towns.

